# P-1222. Oral Cephalexin Population Pharmacokinetics and Target Attainment Analysis in Infants 0-60 Days Old

**DOI:** 10.1093/ofid/ofae631.1404

**Published:** 2025-01-29

**Authors:** Andrew Haynes, Zixuan Wei, Marc H Scheetz, Daniel Gonzalez, Kevin Messacar, Sonya Tang-Girdwood, N Douglas, Peter Anderson

**Affiliations:** University of Colorado School of Medicine, Children's Hospital Colorado, Aurora, Colorado; Skaggs School of Pharmacy and Pharmaceutical Sciences / University of Colorado Anschutz Medical Campus, Aurora, Colorado; Midwestern University, Downers Grove, Illinois; Duke University School of Medicine, Durham, North Carolina; University of Colorado, Children’s Hospital Colorado, Aurora, Colorado; Cincinnati Children's Hospital Medical Center, Fort Thomas, KY; CU Anschutz Skaggs School of Pharmacy and Pharmaceutical Sciences, Aurora, Colorado; Skaggs School of Pharmacy and Pharmaceutical Sciences / University of Colorado Anschutz Medical Campus, Aurora, Colorado

## Abstract

**Background:**

Transitioning from intravenous (IV) to oral (PO) antibiotics is uncommon practice in young infants due to a lack of pharmacokinetic (PK) or pharmacodynamic (PD) data to guide effective PO doses. Cephalexin is a promising PO treatment option against common neonatal pathogens, but rapid renal and intestinal maturation during the first months of life hinder extrapolation of dosing from older populations. In this study, we describe cephalexin’s PK in infants ≤ 60 days old and evaluate dosing regimens to achieve desired PD targets.

**Methods:**

A prospective, single-center, open-label study was conducted to define cephalexin PK parameters in admitted infants 0-60 days old who were either receiving cephalexin clinically or were given a single oral study dose of cephalexin (25 mg/kg). Cephalexin plasma concentrations, quantified using liquid chromatography-mass spectrometry, were used for nonlinear mixed-effects modeling. Monte Carlo simulations were performed to assess the probability of various cephalexin dosing regimens to attain PD targets of free concentrations above the MIC (*f*T > MIC) for 40% and 70% of the day for MICs up to 8 mg/L (assuming 15% protein binding).

**Results:**

The final analysis included 114 cephalexin concentrations obtained from 27 infants [median age: 31 days (range 9-56), gestational age: 37 wks (range 31-41)]. The data were best described using a one-compartment PK model with first order absorption and an absorption lag time. The covariates included in the final PK model were weight on apparent volume of distribution and apparent clearance (CL/F) using standard allometric scaling, post-natal age on absorption rate, and eGFR on CL/F. Cephalexin doses were simulated in 8 cohorts of virtual infants in weekly intervals from 1-8 weeks old assigned normal weights-for-age and eGFRs based on reference values. Conventional cephalexin dosing (25 mg/kg/dose q6 hrs) achieved a 40% *f*T > MIC PD target for every cohort and MIC range, and a 70% *f*T > MIC target in the 1, 2, and 3-week-old cohorts. Higher dosing was needed to achieve 70% *f*T > MIC in infants 4-8 weeks old.

**Conclusion:**

Among infants ≤ 60 days, oral cephalexin can achieve high PD targets for efficacy against common neonatal pathogens with MIC ≤ 8 mg/L. These results provide dosing guidance to facilitate IV to PO transition in young infants.

**Disclosures:**

**Marc H. Scheetz, PharmD, MSc**, Abbvie: Advisor/Consultant|Basilea: Advisor/Consultant|Cidara: Advisor/Consultant|DoseMe: Advisor/Consultant|Entasis: Advisor/Consultant|F2G: Advisor/Consultant|GSK: Advisor/Consultant|Lykos: Advisor/Consultant|Roche: Advisor/Consultant|Third Pole Therapeutics: Advisor/Consultant|Xelia: Advisor/Consultant **Daniel Gonzalez, PharmD, PhD**, Melinta Therapeutics, LLC: Advisor/Consultant|Nabriva Therapeutics plc: Grant/Research Support|Simulations Plus, Inc.: Advisor/Consultant **Sonya Tang-Girdwood, MD, PhD**, Kaizen Biosciences: Advisor/Consultant|Kaizen Biosciences: Grant/Research Support|Pfizer: Grant/Research Support **Peter Anderson, PharmD**, Gilead: Grant/Research Support

